# Identification and in vitro antifungal susceptibility of causative agents of onychomycosis due to *Aspergillus* species in Mashhad, Iran

**DOI:** 10.1038/s41598-021-86038-z

**Published:** 2021-03-24

**Authors:** Xue Xu, Ali Naseri, Jos Houbraken, Farzaneh Akbari, Xiaodong Wang, Rongfen Zhao, Hong Zhang, Mohammad Javad Najafzadeh, Shuwen Deng

**Affiliations:** 1 Department of Medical Microbiology, The People’s Hospital of Suzhou New District, Suzhou, 215000 Jiangsu People’s Republic of China; 2 Department of Parasitology and Mycology, Faculty of Medicine, Mashhad University of Medical Sciences, Mashhad, Iran; 3 Westerdijk Fungal Biodiversity Institute, Utrecht, 3584CT The Netherlands; 4 Cancer Molecular Pathology Research Center, Mashhad University of Medical Sciences, Mashhad, Iran; 5 First Hospital of Xinjiang Medical University, Urumqi, People’s Republic of China

**Keywords:** Evolution, Microbiology, Molecular biology, Diseases, Molecular medicine

## Abstract

*Aspergillus* species are emerging causative agents of non-dermatophyte mold onychomycosis. In this study, 48 *Aspergillus* isolates were obtained from patients with onychomycosis in Mashhad, Iran, during 2015–2018. The aim is to identify the *Aspergillus* isolates to the species level by using partial calmodulin and beta-tubulin gene sequencing and MALDI-TOF MS, and to evaluate their in vitro susceptibility to ten antifungal drugs: terbinafine, itraconazole, voriconazole, posaconazole, ravuconazole, isavuconazole, caspofungin, micafungin, anidulafungin and amphotericin B according to CLSI M38-A3. Our results indicate that *A.flavus* (n = 38, 79%) is the most common *Aspergillus* species causing onychomycosis in Mashhad, Iran. Other detected species were *A. terreus* (n = 3), *A. tubingensis* (n = 2), *A. niger* (n = 1), *A. welwitschiae* (n = 1), *A. minisclerotigenes* (n = 1), *A. citrinoterreus* (n = 1) and *A. ochraceus* (n = 1). *Aspergillus flavus*, *A. terreus* and *A. niger* isolates were correctly identified at the species level by MALDI-TOF MS, while all cryptic species were misidentified. In conclusion, *A. flavus* is the predominant *Aspergillus* species causing onychomycosis due to *Aspergillus* spp. in Mashhad, Iran. MALDI-TOF MS holds promise as a fast and accurate identification tool, particularly for common *Aspergillus* species. It is important that the current database of reference spectra, representing different *Aspergillus* species is expanded to increase the precision of the species-level identification. Terbinafine, posaconazole and echinocandins were i*n vitro* most active against the studies *Aspergillus* isolates *and* terbinafine could be the first choice for treatment of onychomycosis due to *Aspergillus*.

## Introduction

Onychomycosis is a common fungal infection of the nail and is mainly caused by dermatophytes. However, the incidence of onychomycosis due to yeasts and non-dermatophyte molds (NDMs) is increasing^1,2^. *Aspergillus* species have been reported as causal agents of non-dermatophyte mold onychomycosis (NDMO)^2,3^. Bongomin et al. reviewed 42 epidemiological studies globally and found that more than 50% (23/42) of the studies reported *Aspergillus* species as infectious agents of onychomycosis, accounting for 50–100% of the NDMs^4^.

*Aspergillus flavus* is most frequently isolated as causal agent of NMDO in Iran^1,2,5–7^. Due to the non-specific clinical presentation of onychomycosis caused by *Aspergillus*, clinical diagnosis always requires confirmation in a (mycological) laboratory by using direct microscopy, culturing and/or molecular identification^4^. Most of the published studies on NDMO provided diagnosis only based on morphological identification of the isolates, and few reports included data on antifungal activity. In fact, distinguishing closely related *Aspergillus* species based on morphology is difficult or even impossible, whereas a correct identification to species level is critical for optimal antifungal treatment because *Aspergillus* species can have variable drug susceptibility^8^.

Partial calmodulin (*CaM*) and beta-tubulin (*BenA*) gene sequences are currently used for species identification within a given *Aspergillus* species complex^9^. Recent studies have demonstrated that matrix-assisted laser desorption ionization–time of flight mass spectrometry (MALDI-TOF MS) could be an useful alternative for sequence-based identification of *Aspergilli*^10,11^, particularly for phenotypically similar or indistinguishable *Aspergillus* species^11,12^.

In this study, partial *CaM* and *BenA* gene sequences in combination with MALDI-TOF MS analysis are used to identify 48 *Aspergillus* isolates obtained from onychomycosis patients in two hospitals of Mashhad, Iran. Furthermore, antifungal susceptibility testing on those isolates to 10 antifungal drugs including terbinafine is performed using CLSI M38-A3.

## Materials and methods

### Isolation

A total 48 clinical *Aspergillus* isolates were collected from patients diagnosed with onychomycosis during the period from 2015 to 2018 at Emam Reza and Ghaem hospitals in Mashad, Iran. Diagnosis of *Aspergillus* onychomycosis was made based on dystrophic nail appearance and mycological criteria following literature^4^: (1) positive direct microscopy with hyphae presented in nail specimen; (2) positive culture of NDM; (3) repeated culturing of the NDM with absence of dermatophytes and yeasts at different time and samples; (4) molecular identification of the *Aspergillus* isolates.

All specimens were collected using a scalpel and direct microscopic examination was performed using a 20% KOH solution. Sabouraud Glucose Agar (SGA) (Difco, Detroit, MI, USA) containing chloramphenicol (50 mg/L) and cycloheximide (400 mg/L) was used for the isolation of dermatophytes and SGA with chloramphenicol (50 mg/L) for molds. All isolation plates were incubated at 27 °C for up to 4 weeks.

### Sequenced-based and MALDI-TOF MS identification

The isolates were identified by *CaM* and *BenA* gene sequencing^9,13^. The obtained sequences were compared to the NCBI nucleotide database (BLAST; http://blast.ncbi.nlm.nih.gov/Blast.cgi) and the internal sequence database of the Westerdijk Fungal Biodiversity Institute, Utrecht, the Netherlands, containing verified *CaM* and *BenA* gene sequences of all accepted *Aspergillus* species^14^.

MALDI-TOF MS was performed by the formic acid extraction method according to the manufacturer’s instruction (AUTOF MS1000, Autobio, China) and a previous publication with minor modification^15^. All used chemical reagents were of LC–MS grade. Briefly, each *Aspergillus* isolate was cultured on SGA for 3–5 days at 35 °C. After growth, an appropriate amount of sample was collected in a 1.5 mL centrifuge tube containing 1.0 mL 75% ethanol (Sigma-Aldrich, St.Louis, MO, USA). After mixing, the sample was centrifuged at 15,000 × *g* for 5 min and the supernatant discarded. After drying of the residue at 37 °C, 40 µL of lysis solution 1 (containing formic acid) was added and air-dried at room temperature. Subsequently, 1µL was transferred on a target plate, dried naturally in a bio-safety cabinet, and afterwards 1µL of matrix solution was added and dried again. Eventually, for each isolate a mass spectrum was generated and integrated to give a sum spectrum using AUTOF MS1000 (Autobio, China) with in house database.

Species identification by *CaM* and *BenA* gene sequencing and MALDI-TOF MS of 48 *Aspergillus* isolates are shown in Table 1. GenBank accession numbers for *BenA* and *CaM* gene sequences generated in this study are listed in Suppl. Table 1.

**Table 1:** Species identification by *BenA* and *CaM* gene sequencing and MALDI-TOF MS of 48 *Aspergillus* isolates.

Isolate no	Molecular-based ID	MALDI-TOF MS ID
A1	*Aspergillus niger*	*Aspergillus niger*
A2, A4–A6, A8– A9, A11–A24, A26, A28–A31, A33–A34, A38–A39, A41–A48, A50	Aspergillus flavus*	*Aspergillus flavus*
**A3**	***Aspergillus minisclerotigenes***	***Aspergillus flavus***
**A7, A35**	***Aspergillus tubingensis***	***Aspergillus niger***
**A10**	***Aspergillus welwitschiae***	***Aspergillus niger***
A27, A32, A40	*Aspergillus terreus*	*Aspergillus terreus*
**A37**	***Aspergillus citrinoterreus***	***Aspergillus terreus***
A49	*Aspergillus ochraceus*	*Aspergillus ochraceus*

*Molecular identification resulted in *Aspergillus flavus* or *Aspergillus oryzae*. Based on the origin of the isolates, all are identified as *Aspergillus flavus*. The incongruent identifications are given in bold font.

### Antifungal susceptibility testing and statistical analysis

All isolates were tested according to Clinical and Laboratory Standards Institute (CLSI) M38-A3 document^16^. The ten antifungal agents included in this study are itraconazole, voriconazole, posaconazole, ravuconazole, isavuconazole, caspofungin, anidulafungin, amphotericin B (Sigma, Poole, United Kingdom), terbinafine (Aladdin, California, United States) and micafungin (Toronto-Research-Chemicals, Toronto, Ontario, Canada). The antifungal agents were tested at concentrations ranging from 0.008–4 mg/L for echinocandins (micafungin, anidulafungin and caspofungin) and 0.031–16 mg/L for the other compounds. Briefly, all *Aspergillus* isolates were grown on Potato Dextrose Agar (PDA) at 35 °C for 4–5 days to induce sporulation. Conidia were harvested using sterile saline with grinding, and the final inoculum concentration of the suspension was adjusted to 0.4–5 × 10^4^ colony-forming units (CFU) per mL in RPMI 1640 buffered with morpholinepropanesulfonic acid. The plates for echinocandins were incubated at 35 °C for 24 h, and the other plates were incubated at 35 °C for 48 h. Both minimum inhibitory concentrations (MIC) and minimum effective concentrations (MEC) were determined microscopically (Olympus, Japan) at × 40 magnification. *Aspergillus fumigatus* ATCCMYA-3627 and *Candida parapsilosis* ATCC-22019 were used as quality control. MIC/MEC ranges, Geometric mean MIC/MEC, Modal MIC/MEC were calculated for all isolates.

All methods were performed in accordance with the relevant guidelines and regulations as references were given.

### Ethics approval

Ethical approval to conduct the study was obtained from the Ethics Committee of Mashhad University of Medical Sciences (IR.MUMS.MEDICAL.REC.1397.660) and all patients involved understood and agreed to the use of these clinical specimens in the present study.

## Results

### Identification

All 48 isolates were identified based on morphology in combination with BLAST analysis of the generated *BenA* and *CaM* sequences. Gene sequencing revealed presence of eight species (Table 1) belonging to four *Aspergillus* sections, and included four non-cryptic species (*A. flavus*, *A. niger*, *A. terreus* and *A. ochraceus*) and four cryptic species (*A. minisclerotigenes*, *A. citrinoterreus*, *A. tubingensis* and *A. welwitschiae*). Thirty-nine (81%) isolates belonged to section *Flavi* (*A. flavus*, n = 38; *A.minisclerotigenes*, n = 1), four (8%) to section *Terrei* (*A. terreus*, n = 3; *A. citrinoterreus*, n = 1), four (8%) to section *Nigri* (*A. tubingensis*, n = 2; *A. niger*, n = 1; *A. welwitschiae*, n = 1) and one isolate was identified as *A. ochraceus* (section *Circumdati*).

An 89.5% concordance between MALDI-TOF MS and molecular identification was found MALDI-TOF MS allowed the identification of the four common non-cryptic species, but failed to correctly identify the four cryptic species.

### Antifungal susceptibility

The ranges MIC/MEC, Geometric mean MIC/MEC, Modal MIC/MEC, distribution of MICs/MECs of ten antifungal agents against 48 *Aspergillus* isolates are presented in Table 2.

**Table 2:** Ranges MIC/MEC, Geometric mean MIC/MEC, Modal MIC/MEC, distribution of MICs/MECs of 10 antifungal agents against 48 *Aspergillus* isolates.

Species (n)	Antifungal agent	MICs/MECs (mg/L)		No. of isolates with the MIC/MEC (mg/L) of											
		Ranges	MIC_90_/GM	≤ 0.008	0.015	0.03	0.063	0.125	0.25	0.5	1	2	4	8	16
*A.flavus* (38)	Amphotericin B	1–4	2/2.151								5	**24**	9		
	Itraconazole	0.25–1	0.5/0.311						**27**	10	1				
	Voriconazole	0.25–0.5	0.25/0.274						**33**	5					
	Posaconazole	0.03–0.5	0.5/0.097			**13**	9	5		11					
	Isavuconazole	0.125–0.5	0.5/0.274					4	**25**	9					
	Ravuconazole	0.25–0.5	0.5/0.394						13	**25**					
	Anidulafungin	0.008–0.015	0.008/0.008	36	2										
	Caspofungin	0.063–0.25	0.25/0.142				6	**21**	9	2					
	Micafungin	≤ 0.008	0.008/0.008	38											
	Terbinafine	0.03–0.063	0.03/0.03			**35**	3								
*A.minisclerotigenes* (1)	Amphotericin B	1	ND								1				
	Itraconazole	0.25	ND						1						
	Voriconazole	0.25	ND						1						
	Posaconazole	0.5	ND							1					
	Isavuconazole	0.25	ND						1						
	Ravuconazole	0.5	ND							1					
	Anidulafungin	≤ 0.008	ND	1											
	Caspofungin	0.125	ND					1							
	Micafungin	≤ 0.008	ND	1											
	Terbinafine	0.03	ND			1									
*A.niger* (1)	Amphotericin B	1	ND								1				
	Itraconazole	0.5	ND							1					
	Voriconazole	0.25	ND						1						
	Posaconazole	0.5	ND							1					
	Isavuconazole	0.5	ND							1					
	Ravuconazole	0.5	ND							1					
	Anidulafungin	≤ 0.008	ND	1											
	Caspofungin	0.125	ND					1							
	Micafungin	≤ 0.008	ND	1											
	Terbinafine	0.03	ND			1									
*A. welwitschiae* (1)	Amphotericin B	0.5	ND							1					
	Itraconazole	0.5	ND							1					
	Voriconazole	0.25	ND						1						
	Posaconazole	0.5	ND							1					
	Isavuconazole	0.25	ND						1						
	Ravuconazole	0.25	ND						1						
	Anidulafungin	≤ 0.008	ND	1											
	Caspofungin	0.125	ND					1							
	Micafungin	≤ 0.008	ND	1											
	Terbinafine	0.03	ND			1									
*A.tubingensis* (2)	Amphotericin B	0.25–1	ND						1		1				
	Itraconazole	1	ND								**2**				
	Voriconazole	0.25	ND						**2**						
	Posaconazole	0.25–1	ND						1		1				
	Isavuconazole	0.5–1	ND							1	1				
	Ravuconazole	0.5–1	ND							1	1				
	Anidulafungin	≤ 0.008	ND	**2**											
	Caspofungin	0.063–0.125	ND				1	1							
	Micafungin	≤ 0.008	ND	**2**											
	Terbinafine	0.03–0.5	ND			1				1					
*A. terreus* (3)	Amphotericin B	0.5–1	1/0.794							1	**2**				
	Itraconazole	0.25–0.5	0.5/0.397						1	**2**					
	Voriconazole	0.25–0.5	0.5/0.315						**2**	1					
	Posaconazole	0.063–0.125	0.125/0.0792162				**2**	1							
	Isavuconazole	0.25–0.5	0.5/0.397						1	**2**					
	Ravuconazole	0.25–0.5	0.5/0.397						1	**2**					
	Anidulafungin	≤ 0.008	0.008/0.008	**3**											
	Caspofungin	0.25	0.25/0.25						**3**						
	Micafungin	≤ 0.008	0.008/0.008	**3**											
	Terbinafine	0.25	0.25/0.25						**3**						
*A.citrinoterreus* (1)	Amphotericin B	4	ND										1		
	Itraconazole	0.25	ND						1						
	Voriconazole	0.25	ND						1						
	Posaconazole	0.03	ND			1									
	Isavuconazole	0.125	ND					1							
	Ravuconazole	0.125	ND					1							
	Anidulafungin	≤ 0.008	ND	1											
	Caspofungin	0.25	ND						1						
	Micafungin	≤ 0.008	ND	1											
	Terbinafine	0.25	ND						1						
*A. ochraceus* (1)	Amphotericin B	2	ND									1			
	Itraconazole	0.25	ND						1						
	Voriconazole	0.5	ND							1					
	Posaconazole	0.03	ND			1									
	Isavuconazole	0.5	ND							1					
	Ravuconazole	0.5	ND							1					
	Anidulafungin	≤ 0.008	ND	1											
	Caspofungin	0.25	ND						1						
	Micafungin	≤ 0.008	ND	1											
	Terbinafine	0.03	ND			1									

Abbreviations: minimum inhibitory concentration, MIC;minimum effective concentration, MEC; values in bold indicate modal or most frequent MICs, Modal MIC/MEC; MICs are shownfor amphotericin B, itraconazole, posaconazole, voriconazole, ravuconazole, isavuconazole; MECs are shown for micafungin, caspofungin and anidulafungin.ND: not determined.

In general, among 48 *Aspergillus* isolates tested in this study, the lowest Modal MIC/MEC (< 0.008 mg/L) were those of anidulafungin and micafungin, followed by terbinafine and posaconazole (0.031 mg/L). The other four azole compounds tested were active in vitro, with MICs of ≤ 1 mg/L. Posaconazole and caspofungin revealed variable MIC/MEC values with MIC ranges from 0.031 to 0.5 mg/L and MEC 0.063–0.25 mg/L, respectively.

The 38 *A. flavus* isolates showed relatively high MIC value to amphotericin B (range: 1–4 mg/L, Modal MIC: 2 mg/L, n = 24), but lower MICs than the epidemiological cutoff value (ECV) (1 mg/L) for all azoles tested. When testing the susceptibility to terbinafine, all *A. flavus* isolates revealed low MIC with Modal MIC 0.031 mg/L (n = 35). A similar MIC value is observed for the *A. minisclerotigenes* isolate*.*

For section *Terrei*, three *A. terreus* isolates had a similar antifungal susceptibility profile as *A. flavus*, except terbinafine, which has higher MICs (Modal MIC 0.25 mg/L, n = 3). In contrast to *A. terreus*, the isolate of the cryptic species *A. citrinoterreus* had a MIC of 4 mg/L to amphotericin B.

All section *Nigri* isolates revealed lower MIC and Modal MIC ≤ 1 mg/L to the five azoles tested. The majority isolates of section *Nigri* have an MIC value of 0.031 mg/L to terbinafine except one *A. tubingensis* isolate with an MIC value of 0.5 mg/L.

## Discussion

This study presented, for the first time, an overview of the occurrence of *Aspergillus* species causing onychomycosis in Mashhad, Iran, including a molecular characterization of the isolates and in vitro susceptibility to 10 antifungal agents. In total 48 *Aspergillus* isolates were obtained from nail in patients with onychomycosis in two medical centers of Mashhad, Iran.

Our study highlights the epidemiological features that *A. flavus* is the predominant species causing NDMO due to *Aspergillus*. in Mashhad, Iran. This finding is similar to those reported from other places in Iran^1,6,7,17,18^ and differs from Sri Lanka^19^, Cameroon^20^, India^21,22^, Turkey^23^ where *A. niger* is the most frequently species, and from Italy^24^ and UK^25^ where *A. fumigatus* and *A. terreus* are the most common agents, respectively. Data from Iran suggest that *A. flavus* is most common agent involved in NDMO^5–7^. Besides onychomycosis, *A. flavus* is also the leading cause of chronic fungal rhinosinusitis in Iran^26^. This could be due to the arid climate in Iran that favors the growth of thermo-tolerant fungi like *A. flavus*. *Aspergillus tubingensis* and *A. welwitschiae* (former name *A. awamorii*) in section *Nigri* have previously been reported as emerging causal agents of onychomycoses^2,27^. Both species have been reported as the causal agents of otomycoses as well^28–30^. In our study, four cryptic species were identified based on the analysis of *BenA* and *CaM* gene sequences: *A. minisclerotigenes* in section *Flavi*, *A. citrinoterreus* in section *Terrei* and *A. tubingensis* and *A. welwitschiae* in section *Nigri*. To our knowledge, *A. minisclerotigenes*, *A. citrinoterreus* and *A. ochraceus* are for the first time identified as causative agents of NDM onychomycosis. *Aspergillus minisclerotigenes*is is closely related to *A. flavus*^31^. Dehghan et al.^32^ reported a human infection caused by *A. minisclerotigenes* from Iran in 2014 and Esfahani et al*.*^33^ reported the first case of fungal keratitis due to this species in Iran in 2019, suggesting that the occurrence of *A. minisclerotigenes* might have been underreported due to identification based on morphology. The recently described *A. citrinoterreus* within section *Terrei*^34^ has been reported as causative agent involved inhuman invasive aspergillosis^35–37^. *Aspergillus ochraceus* belongs to *Aspergillus* section *Circumdati*. This species has rarely been related to human infection^38,39^. So far, only two cases reported *A. ochraceus* as a pathogenone case was an invasive pulmonary aspergillosis from Poland^38^, another case was allergic bronchopulmonary aspergillosis^39^.

In our study, 38 isolates were molecularly identified as *A. flavus* or *A. oryzae*. These two species are genetically very similar and indistinguishable by *BenA* and *CaM* gene sequencing^40^. Identification of these species is based on the origin and/or toxin production potential: *A. oryzae* is used in (food) fermentations and biotechnology and does not produce aflatoxins, while *A. flavus* is not domesticated and can produce aflatoxins, though non-toxigenic strains also occur^41^.Of note, all 38 *A. flavus* isolates were corrected identification by MALDI-TOF MS analysis based on the AUTOF MS 1000 (Autobio, China) in-house database. Similarly, De Carolis et al.^40^ reported that MALDI-TOF MS easily differentiated *A. flavus* and *A.oryzae* on the species level. In contrast to some investigators^42,43^, who reported that only 18 of 200 isolates were confirmed as *A. flavus* using MALDI-TOF MS based on the Bruker score database among a set of 200 clinical and environmental *A. flavus* isolates identified by sequencing. Masih et al. ^43^ also showed that *A. oryzae* can easily be identified as *A. flavus*. Those discrepancy could be due to differences between MALDI-TOF MS machines, the species coverage in reference databases, interpretive cutoffs and methodology applied for sample preparation^11^.

In our study, *A. minisclerotigenes* was misidentified as *A. flavus* by MALDI-TOF MS*.* Similarly, *A. tubingensis* and *A. welwitschiae* were misidentified as *A. niger* and *A. citrinoterreus* was misidentified as *A. terreus*. These are due to the inadequate number of reference spectra in the used in-house database (Suppl. Table 2). Therefore, databases should be expanded with well-validated spectra of cryptic species in order to get an accurate identification of all (clinical relevant) *Aspergillus* species. Our study confirms the discriminatory power of MALDI-TOF MS for common clinical *Aspergillus* species^40^.

The antifungal susceptibility data generated in this study indicate that terbinafine has excellent in vitro activity against the eight species tested. Terbinafine appears more active against isolates of section *Flavi* (GM 0.032 mg/L and section *Nigri* (GM 0.077 mg/L) than to section *Terrei* isolates (GM 0.25 mg/L), and has superior activity against *A. flavus* compared to the azoles and amphotericin B*.* Similar results are obtained in previous studies^44,45^. Thus, terbinafine could be placed as an alternative drug for treatment of onychomycosis caused by *Aspergillus* species with the confirmation of clinical efficacy of terbinafine in the management of onychomycosis due to *Aspergillus* spp.^4^.

Based on the proposed epidemiologic cut-off values (ECV) of *A. flavus* (posaconazole 0.5 mg/L; itraconazole 1 mg/L; voriconazole 1 mg/L; isavuconazole1 mg/L and amphotericin B 4 mg/L)^46^, all azoles tested in our study exhibited good activity to 38 *A. flavus* isolates and this is in agreement with previous reports^18,19^. Posaconazole had the lowest GM value (0.097 mg/L), followed by voriconazole and isavuconazole (both 0.274 mg/L), itraconazole and ravuconazole (0.311 and 0.394 mg/L, respectively) in an increasing order. Although no antifungal susceptibility ECV are available for *A. flavus* to ravuconazole, in our study, all *A. flavus* isolates had MIC values less than 1 mg/L (MIC range 0.25–0.5 mg/L), indicating the potency of this antifungal against *A. flavus,* which is in agreement with those reported by Pfaller et al.^47^. Amphotericin B showed relatively high MICs (GM 2.151 mg/L; MIC range 1–4 mg/L) against all *A. flavus* isolates, including a MIC of 4 mg/L for nine (23.6%) isolates of *A. flavus*, which is similar to previous reports from the United States, Europe^48,49^, and the Middle East^50,51^. The *A. minisclerotigenes* isolate had similar MIC values as *A. flavus*.

Among the section *Nigri* isolates, the azoles tested were active against all isolates, although an *A. tubingensis* isolate presented a slightly higher MIC value for azoles, which is similar to results reported previously^52^.

The three *A. terreus* and one *A. citrinoterreus* isolate displayed low MICs for the tested azoles. Posaconazole was the most effective azole against *A. terreus* (modal MIC 0.063 mg/L, n = 2) which is similar to previous studies^53,54^.

Although A *terreus* is intrinsically resistant to amphotericin B, 12–13% of the isolates have low amphotericin B MICs^55,56^. Our results also show that the three *A. terreus* isolates exhibited MICs < 2 mg/L, which is below the proposed ECV (MIC 4 mg/L). However, the *A. citrinoterreus* isolate had a MIC of 4 mg/L for amphotericin B, which is in agreement with those reported by Imbert et al.^35^.

Echinocandins showed good activity against the most *Aspergillus* isolates in this collection, while anidulafungin and micafungin showed a lowest Modal MEC of 0.008 mg/L, followed by caspofungin with Modal MEC of 0.125–0.5 mg/L. Anidulafungin and micafungin appeared more potent than caspofungin and this is consistent with several previous studies^44,57^. Of note, the four section *Terrei* isolate have higher MEC values (0.5 mg/L), above ECV (0.125 mg/L). The similar results were reported by Lass-Flörl et al*.* With a set of 48 clinical and 31 environmental *A. terreus* isolates, they showed that caspofungin has higher MECs (MEC_90_ 2 mg/L) than anidulafungin (MEC_90_ 0.03 mg/L) and micafungin (MEC_90_ 0.02 mg/L)^58^.

## Conclusion

Based on molecular and MALDI-TOF MS identification, *A. flavus* (79%) is the most common *Aspergillus* species in NDM onychomycosis due to *Aspergillus* in Mashhad, Iran. The other isolates showed a wider species diversity. We emphasize the importance of using molecular methods to accurately identify *Aspergillus* at the species level because different species may vary in terms of susceptibility to antifungal agents. However, our results are limited by the relatively low number of clinical *Aspergillus* isolates obtained in Mashhad, Iran. Terbinafine, posaconazole, and echinocandins are shown in vitro to be the most potent antifungal agents against *Aspergillus* spp. Terbinafine could be first line drug for treatment of onychomycosis due to *Aspergillus*, the in vivo efficacy remains to be determined.

## Supplementary Information

GenBank accession numbers for generated *BenA* and *CaM* genesequences.

The represented species of the genus*Aspergillus* in the in housedatabase of AUTOF MS 1000 (Autobio, China).

## Data Availability

The samples utilized in our study were obtained from preexisting samples isolated from patients who routinely referred to the hospital for direct examination and culture in Clinical Lab. And we used the culture for this research, and no additional samples were taken from the patient.
