# Supplementary material for: Identification and in vitro antifungal susceptibility of causative agents of onychomycosis due to Aspergillus species in Mashhad, Iran
Source: Sci Rep. 2021 Mar 24;11:6808. doi: 10.1038/s41598-021-86038-z (PMC7991633; doi:10.1038/s41598-021-86038-z)
Supplement: Supplementary file 1 — GenBank accession numbers for generated BenA and CaM genesequences. [file 41598_2021_86038_MOESM1_ESM.docx]

| **Suppl. Table 1.GenBank accession numbers**  **for generated *BenA* and*CaM* gene sequences** | | | |
| --- | --- | --- | --- |
| Isolate no. | Species name | Accession no. (*BenA*) | Accession no. (*CaM*) |
| A1 | *Aspergillus niger* | MT842885 | MT842894 |
| A2 | *Aspergillus flavus* | MT842846 | – |
| A3 | *Aspergillus minisclerotigenes* | MT842847 | MT842895 |
| A4 | *Aspergillus flavus* | MT842848 | MT842896 |
| A5 | *Aspergillus flavus* | MT842849 | MT842897 |
| A6 | *Aspergillus flavus* | MT842850 | MT842898 |
| A7 | *Aspergillus tubingensis* | MT842887 | MT842899 |
| A8 | *Aspergillus flavus* | MT842851 | MT842900 |
| A9 | *Aspergillus flavus* | MT842852 | MT842901 |
| A10 | *Aspergillus welwitschiae* | MT842886 | MT842902 |
| A11 | *Aspergillus flavus* | MT842853 | MT842903 |
| A12 | *Aspergillus flavus* | MT842854 | MT842904 |
| A13 | *Aspergillus flavus* | MT842855 | MT842905 |
| A14 | *Aspergillus flavus* | MT842856 | – |
| A15 | *Aspergillus flavus* | MT842857 | MT842906 |
| A16 | *Aspergillus flavus* | MT842858 | MT842907 |
| A17 | *Aspergillus flavus* | MT842859 | MT842908 |
| A18 | *Aspergillus flavus* | MT842860 | MT842909 |
| A19 | *Aspergillus flavus* | MT842861 | – |
| A20 | *Aspergillus flavus* | MT842862 | MT842910 |
| A21 | *Aspergillus flavus* | MT842863 | MT842911 |
| A22 | *Aspergillus flavus* | MT842864 | MT842935 |
| A23 | *Aspergillus flavus* | MT842865 | MT842912 |
| A24 | *Aspergillus flavus* | MT842866 | MT842913 |
| A26 | *Aspergillus flavus* | MT842867 | MT842914 |
| A27 | *Aspergillus terreus* | MT842889 | MT842915 |
| A28 | *Aspergillus flavus* | MT842868 | – |
| A29 | *Aspergillus flavus* | MT842869 | MT842916 |
| A30 | *Aspergillus flavus* | MT842870 | MT842917 |
| A31 | *Aspergillus flavus* | MT842871 | MT842918 |
| A32 | *Aspergillus terreus* | MT842890 | MT842919 |
| A33 | *Aspergillus flavus* | MT842872 | MT842920 |
| A34 | *Aspergillus flavus* | MT842873 | MT842921 |
| A35 | *Aspergillus tubingensis* | MT842888 | MT842922 |
| A37 | *Aspergillus citrinoterreus* | MT842892 | MT842923 |
| A38 | *Aspergillus flavus* | MT842874 | MT842924 |
| A39 | *Aspergillus flavus* | MT842875 | – |
| A40 | *Aspergillus terreus* | MT842891 | MT842925 |
| A41 | *Aspergillus flavus* | MT842876 | MT842926 |
| A42 | *Aspergillus flavus* | MT842877 | MT842927 |
| A43 | *Aspergillus flavus* | MT842878 | MT842928 |
| A44 | *Aspergillus flavus* | MT842879 | MT842929 |
| A45 | *Aspergillus flavus* | MT842880 | MT842930 |
| A46 | *Aspergillus flavus* | MT842881 | MT842931 |
| A47 | *Aspergillus flavus* | MT842882 | MT842932 |
| A48 | *Aspergillus flavus* | MT842883 | MT842933 |
| A49 | *Aspergillus ochraceus* | MT842893 | – |
| A50 | *Aspergillus flavus* | MT842884 | MT842934 |
