# Supplementary material for: Identification and in vitro antifungal susceptibility of causative agents of onychomycosis due to Aspergillus species in Mashhad, Iran
Source: Sci Rep. 2021 Mar 24;11:6808. doi: 10.1038/s41598-021-86038-z (PMC7991633; doi:10.1038/s41598-021-86038-z)
Supplement: Supplementary file 2 — The represented species of the genusAspergillus in the in housedatabase of AUTOF MS 1000 (Autobio, China). [file 41598_2021_86038_MOESM2_ESM.docx]

**Suppl. Table 2. The represented species of the genus *Aspergillus* in the in-house**

| **database of the AUTOF MS 1000 (Autobio, China)** | |
| --- | --- |
| No. | Species name |
| 1 | *Aspergillus brasiliensis* |
| 2 | *Aspergillus candidus* |
| 3 | *Aspergillus clavatus* |
| 4 | *Aspergillus flavus* |
| 5 | *Aspergillus fumigatus* |
| 6 | *Aspergillus glaucus* (syn. *Eurotium herbariorum*) |
| 7 | *Aspergillus* *montevidensis (*syn. *A. amstelodami, Eurotium amstelodami*) |
| 8 | *Aspergillus nidulans* (syn. *Emericella nidulans*) |
| 9 | *Aspergillus niger* |
| 10 | *Aspergillus ochraceus* |
| 11 | *Aspergillus oryzae* |
| 12 | *Aspergillus parasiticus* |
| 13 | *Aspergillus sydowii* |
| 14 | *Aspergillus tamarii* |
| 15 | *Aspergillus terreus* |
| 16 | *Aspergillus thermomutatus* (syn. *Neosartorya pseudofischeri*) |
| 17 | *Aspergillus unguis* |
| 18 | *Aspergillus ustus* |
| 19 | *Aspergillus versicolor* |
